# Increased proton pump inhibitor and NSAID exposure in irritable bowel syndrome: results from a case-control study

**DOI:** 10.1186/1471-230X-12-121

**Published:** 2012-09-05

**Authors:** Daniel Keszthelyi, Gwen H Dackus, Gwen M Masclee, Joanna W Kruimel, Ad AM Masclee

**Affiliations:** 1 Top Institute Food and Nutrition, Wageningen, The Netherlands; 2 Division of Gastroenterology-Hepatology, Department of Internal Medicine, Maastricht University Medical Centre+, Maastricht, The Netherlands

**Keywords:** Irritable bowel syndrome, Proton pump inhibitors, NSAIDs, Small intestinal bacterial overgrowth, Intestinal permeability

## Abstract

**Background:**

Patients with irritable bowel syndrome (IBS) seen by a gastroenterologist often utilize medications that may alter intestinal homeostasis. The question arises whether exposure to these drugs is associated with the development of IBS symptoms. Aim of this study was therefore to assess the use of PPIs and NSAIDs in patients with IBS versus controls.

**Methods:**

Cases of IBS from the last 5 years were reviewed. All patients having had at least one prescription for a particular drug (PPIs, NSAIDs, SSRIs, diuretics, ACE inhibitors) in the 6 months prior to the time of initial symptom onset were considered exposed. The control group consisted of individuals randomly selected from the general population.

**Results:**

287 cases of IBS were retrieved for analysis together with 287 age and sex-matched controls. Exposure to PPIs and NSAIDs was significantly higher in IBS patients, whereas no association between ACE inhibitor use and IBS was found. PPIs were not significantly associated when excluding patients with gastrointestinal reflux disease or functional dyspepsia. Exposure to SSRIs was also positively associated with IBS, but only when patients with psychiatric comorbidity were included in the analyses.

**Conclusions:**

Medications that may alter intestinal homeostasis such as NSAIDs and PPIs were more frequently used in IBS patients compared to controls. This association might be relevant for everyday clinical practice, but it is remains to be elucidated whether this association is of etiological nature.

## Background

Irritable bowel syndrome (IBS) is a condition characterized by recurrent abdominal pain or discomfort accompanied by abnormalities in bowel habits [1]. It leads to considerable morbidity as it is associated with tripling of missed work-days and doubling of illness-related costs [1]. National Health Service costs in the UK are 60% higher in IBS patients than in non-IBS control patients [2]. As care-seeking behavior can be characteristic for IBS patients, these patients are also prone to suffer from polyphragmasia and polypharmacia.

Since the pathogenesis of IBS is still poorly understood, the question arises whether the intake of medication can contribute to or trigger onset of symptoms in IBS patients, as these patients often utilize medications that may potentially alter intestinal homeostasis. One postulated hypothesis points to increased intestinal permeability in IBS patients induced by exposure to certain medication, such as non-steroidal anti-inflammatory drugs (NSAIDs), thereby allowing luminal antigens to enter the lamina propria and eliciting an immune and inflammatory reaction [3]. In particular, proton pump inhibitors (PPIs) have also been suggested to be involved in the pathogenesis of IBS, primarily through alteration of intestinal microbiota composition [4]. PPIs are one of the most frequently prescribed classes of medication worldwide because they combine a high level of efficacy with low toxicity. In 2009, expenses for omeprazole and esomeprazole only reached €150 million in the Netherlands [5]. In the five years since the introduction of esomeprazole in 2001, prescriptions for PPIs have doubled [6]. With 5 million prescriptions, omeprazole became the second most frequently prescribed medication in the Netherlands in 2009 [5]. A recent socio-demographic study from the Netherlands found that 11.8% of the general population had at least one prescription for a PPI in the year 2006 [7]. It has also been demonstrated that in IBS patients, the use of PPIs generates an excess health cost of €80 per case per year [8].

We have recently demonstrated that exposure to PPIs was associated with increased risk for developing microscopic colitis [9], a condition known to share symptoms and clinical features with IBS [10] and to be associated with changes in intestinal permeability [11]. PPIs have not only been demonstrated to be able to alter intestinal barrier function [12], but also to induce profound changes in intestinal microbiota composition [13], which in turn may also lead to secondary changes in epithelial integrity [14]. It has therefore also been hypothesized that PPIs use is associated with IBS through causing small intestinal bacterial overgrowth (SIBO) [4]. Due to these presumed alterations in intestinal physiology induced by PPI and/or NSAID use, we postulate that PPIs and NSAIDs may play an etiological role in the development of IBS. More specifically, we hypothesize that patients with IBS will be more often exposed to drugs affecting intestinal homeostasis (PPIs and NSAIDs) at time of presentation with symptoms. In this study, we aimed to establish a relationship between PPI or NSAID use and IBS by comparing exposure these drugs in comparison to control group from the general population.

## Methods

The study was approved by the Medical Ethics Committee of the Maastricht University Medical Centre+ (MUMC), Maastricht, the Netherlands (reference number 0904015), and performed in full accordance with the European directive 91/507/EEG, and the Declaration of Helsinki (as amended in Tokyo, Venice, Hong Kong, Somerset West and Edinburgh. Note of clarification added in Washington and Tokyo).

### Patients

We performed a retrospective, observational, case–control study at the Division of Gastroenterology-Hepatology at the Maastricht University Medical Center, the Netherlands, a tertiary and regionally also secondary referral medical center. Patients presenting with symptoms characteristic for IBS who had been referred to a gastroenterologist at our outpatient clinic by general practitioners or other medical specialists for diagnostic work up and therapy were identified by reviewing medical records in the period from May 2006 to November 2010. Symptoms included abdominal pain and discomfort associated with diarrhea or constipation, bloating and abdominal distension. At the time of the index visit to our outpatient clinic, the duration of the symptoms was assessed. In case of patients having had symptoms for at least 6 months, the diagnosis of IBS was made based on Rome III criteria at the time of the index visit. In case of patients having IBS symptoms for less than 6 months, diagnosis according to Rome III criteria was confirmed using questionnaires sent to patients to assess symptom duration following initial symptom onset. Only patients fulfilling Rome III criteria were included in the investigation.

Written informed consent was obtained from patients to obtain detailed information of their pharmacy records. Their medical records were reviewed to assess medical history and comorbidities. Information on prescribed medication for the period of two years prior to the index visit was obtained from the pharmacy database. All patients having had at least one prescription for a period of four weeks for a PPI (esomeprazole, omeprazole, pantoprazole, lansoprazole or rabeprazole) in a dose of 20 mg (for lansoprazole 15 mg) or more in the 180 days prior to the time of the initial onset of symptoms were considered exposed to PPIs. Patients exposed to PPIs only in the period *after* initial onset of symptoms were not included in the analyses. Similarly, we investigated exposure to NSAIDs (as drugs known to alter intestinal barrier function). The following doses were considered as minimal drug exposure: diclofenac 12.5 mg, ibuprofen 200 mg, ketoprofen 100 mg, indomethacin 25 mg, aceclofenac 100 mg, nabumetone 500 mg, naproxen 250 mg, aspirin 500 mg. COX-2 inhibitors were not included in the analyses. Also, exposure to selective serotonin reuptake inhibitors (SSRIs, drugs frequently prescribed to IBS patients) and as control medication exposure to diuretics and angiotensin converting enzyme (ACE) inhibitors, drugs that have not been associated with IBS, was investigated. The rationale for selecting a time window of 180 days between drug exposure and diagnosis of IBS was the minimum of six months duration of symptoms needed for IBS diagnosis (Rome III criteria) and the considerable delay that may occur between initial symptom onset and referral with eventual diagnosis by a gastroenterologist. Psychiatric comorbidity was defined as evidence of depression or anxiety disorder in medical history as diagnosed according to the DSM IV.

### Controls

The control group consisted of 408 individuals randomly selected from the general population in Maastricht, the Netherlands. Residents with a permanent address in Maastricht were eligible for selection. Potential controls were selected by a random computerized selection from the municipality residential register. Controls received questionnaires regarding their current medical status and drug exposure. Individuals who self-admitted to being diagnosed with IBS were excluded. Controls for analyses (n = 287) were selected from this group and matched to IBS cases by age (within 1 year) and gender.

### Statistical analysis

Statistical analyses were performed using χ^2^ test to compare gender and comorbidities. Independent Student’s t test was used to compare age and BMI. Generalized linear model for binomial regression also adjusted for comorbidities (psychiatric, gastrointestinal reflux disease [GERD], functional dyspepsia [FD], rheumatoid disorders, fibromyalgia) was used to calculate ORs and 95% CIs using SPSS, version 20.0 (Chicago, IL). Statistical significance was predetermined as p < 0.05.

## Results

During the investigated period, 521 cases were identified as having IBS according the Rome III criteria. From these, a total of 287 cases gave informed consent to assess their pharmacy records. These cases of IBS along with 287 randomly selected age and sex-matched controls were identified and retrieved for detailed analysis. For demographic characteristics, see Table 1. Distribution of IBS subtypes was as follows: 38% diarrhea-predominant (IBS-D), 30% constipation-predominant (IBS-C) and 32% mixed subtype (IBS-M). A significantly higher numer of IBS had comorbid conditions (FD or GERD, psychiatric condition, fibromyalgia, rheumatoid arthritis), compared to controls (see Table 2, all p < 0.001).

**Table 1:** Demographic characteristics of irritable bowel syndrome (IBS) patients and controls

	**IBS patients N = 287**	**Controls N = 287**	**P value**
Age (years)	36.7 ± 16	37.4 ± 16	0.64
Gender	75% female	75% female	1.00
BMI (kg/m^2^)	25.8 ± 3.4	24.7 ± 5.1	0.06

**Table 2:** Results for binary logistic regression analysis using comorbidities and exposure to drugs in IBS

	**IBS patients N = 287**	**Controls N = 287**	**OR [95% CI] uncorrected for comorbidities**	**OR [95% CI] corrected for comorbidities**	**OR [95% CI] excluding patients with psychiatric comorbidity**	**OR [95%] excluding patients with RA or arthritis**	**OR [95% CI] excluding patients with GERD/FD**
FD or GERD	25.2%	9.8%	NA	2.0 [1.1-3.5]	2.8 [1.5-5.2]	2.1 [1.8-3.8]	NA
Psychiatric comorbidity	40.4%	3.5%	NA	16.6 [7.9-34.8]	NA	17.0 [8.0-36.2]	33.3 [11.6-95.5]
Fibromyalgia	4.4%	0%	NA	NA	NA	NA	NA
Rheumatoid arthritis	6.1%	0%	NA	NA	NA	NA	NA
PPIs	21.2%	5.2%	2.2 [1.1-5.0]	2.1 [1.1-4.1]	3.0 [1.3-7.0]	3.1 [1.5-6.2]	2.1 [0.8-5.4]
NSAIDs	20.55%	3.8%	3.8 [1.7-8.6]	5.2 [2.5-11.0]	4.1 [1.7-9.6]	5.7 [2.65-12.2]	5.5 [2.3-13.3]
SSRIs	10.84%	2.1%	3.7 [1.3-10.9]	0.9 [0.3-2.7]	6.3 [0.7-56.8]	0.75 [0.25-2.3]	2.8 [0.5-14.7]
Diuretics	5.2%	2.8%	1.0 [0.3-3.6]	1.1 [0.3-3.4]	2.4 [0.7-7.9]	1.4 [0.5-3.7]	2.6 [0.8-9.6]
ACE-I	2.8%	1.4%	1.2 [0.2-4.0]	1.4 [0.3-6.0]	0.9 [0.2-4.4]	1.3 [0.3-5.2]	0.8 [0.1-5.0]

PPIs = proton pump inhibitors PPIs, NSAIDs = non-steroidal anti-inflammatory drugs, SSRIs = selective serotonin reuptake inhibitors, ACE-I = angiotensin-converting enzyme inhibitors, RA = rheumatoid arthritis, GERD = gastroesophageal reflux disease, FD = functional dyspepsia. NA = not applicable.

Exposure to PPIs and NSAIDs was significantly higher in IBS patients (see Table 2). Co-exposure to PPIs and NSAIDs was also significantly higher in IBS (7.6% vs 0%, χ^2^ = 17.4, p < 0.001). Of the patients using NSAIDs (59/287), 37% also used PPIs at the same time (22/287). See Table 3 for a summary of the type of PPIs and NSAIDs patients were exposed to.

**Table 3:** Types of drugs used by irritable bowel syndrome patients

**Type of PPI**^**1**^		**Type of NSAID**^**2**^	
Pantoprazole	44.0%	Diclofenac	47.0%
Omeprazole	28.8%	Ibuprofen	41.2%
Esomeprazole	27.1%	Naproxen	21.6%
Rabeprazole	6.7%	Aceclofenac	3.9%

^1^ 6 patients used multiple PPIs.

^2^ 7 patients used multiple NSAIDs.

PPI = proton pump inhibitor. NSAID = non-steroidal anti-inflammatory drug.

IBS patients more frequently used SSRIs (Table 2). However, no association with SSRI use was found when correcting for psychiatric comorbidity. When patients with psychiatric co-morbidity were excluded from analysis, ORs for PPIs, NSAIDs diuretics and ACE inhibitors remained unchanged, whereas SSRI exposure proved not to be associated with IBS (Table 2).

No association between ACE inhibitor or diuretic use and IBS was found (Table 2). No significant association was found with regards to IBS-subtype (diarrhea, constipation or mixed) and drug exposure.

As PPIs are most frequently prescribed for upper GI tract complaints originating primarily from FD or GERD, analyses were repeated with exclusion of patients and controls with documented comorbid GERD or dyspepsia. These analyses showed similar ORs for NSAIDs, SSRIs, diuretics and ACE inhibitors. However, PPI exposure proved not be associated with the disorder in the group of IBS patients without comorbid GERD or dyspepsia (OR 2.1 [0.8-5.4]).

Similarly, as NSAIDs are often prescribed for rheumatic disorders, analyses were also conducted with the exclusion of patients and controls with documented rheumatoid arthritis. These analyses showed similar ORs to the analysis with the inclusion of all subjects.

## Discussion

In this study, we found that a significantly higher number of IBS patients use certain medications that can potentially alter intestinal homeostasis, such as PPIs and NSAIDs, in comparison to controls. Medication use of patients was examined in the period preceding the time of presentation at our clinic with their IBS symptoms, aiming to establish a temporal relationship between medication use and onset of symptoms.

We here demonstrate in our study population of IBS patients a positive association with IBS and exposure to PPIs and NSAIDs, but also to SSRIs. The former two are associated with alterations of intestinal physiology, whereas the latter is often prescribed in IBS due to concomitant psychiatric comorbidity. In order to delineate between a potential causal effect and mere therapeutic utilization of these drugs, we conducted analysis with the exclusion of IBS patients with certain comorbid conditions. For instance, IBS patients often suffer from functional dyspepsia; the degree of overlap varies between 15 and 42% [15,16]. Because patients with IBS are more likely to have GERD and dyspepsia in comparison to controls, they are also more likely to receive PPI therapy. Therefore, following initial analyses, we conducted additional analyses excluding the cases and controls exposed to PPIs due to therapy for concomitant upper GI complaints based on functional dyspepsia or GERD. In this case, PPI exposure was not associated with IBS. We therefore presume that the exposure to PPIs can be explained by the therapeutic use of PPIs for upper GI complaints.

We also found a positive association with the exposure to NSAIDs, reproducing previous literature findings [17]. Pain-related symptoms of extragastrointestinal origin frequently observed in IBS could be the explanation for the increased use of NSAIDs in this population [18]. Moreover, as IBS patients tend to suffer from recurring and therapy-refractory abdominal pain, overuse of NSAIDs in IBS patients can be extremely common in everyday clinical practice. Therefore, despite the fact that NSAIDs are known to alter intestinal physiology [19] and in particular barrier function, previous studies suggest that the association with NSAIDs is due to the tendency of patients with IBS having pain complaints rather than analgesics being a causative factor [17]. We therefore conducted analyses with the exclusion of subjects with rheumatoid disorders, as a common source of extragastrointestinal pain. Results of these analyses still showed a significant association between NSAIDs use and IBS. This could be explained by the fact that patients were taking NSAIDs due to pain symptoms related to conditions other than rheumatoid disorders. On the other hand, recent findings have shown that NSAIDs can compromise intestinal permeability in IBS patients to a greater extent than in healthy subjects [3]. Furthermore, another epidemiological study demonstrated that IBS patients using NSAIDs were also more likely to have persistent irritable bowel syndrome [20]. This appears to be in line with the hypothesis that NSAID therapy affects intestinal permeability resulting in sustained low-grade mucosal inflammation [21]. This implies that even if NSAIDs do not necessarily trigger IBS symptoms, they may be able to sustain the condition by altering intestinal physiology and in particular by impairing intestinal permeability.

PPIs and NSAIDs are often used simultaneously, with the former frequently co-prescribed to reduce gastrointestinal injury due to the latter. In our study, over a third of the patients using NSAIDs also used PPIs – presumably for gastroprotection. Recent video capsule studies suggest [22,23] a very high incidence (55-70%) of intestinal damage in healthy humans taking both NSAIDs and PPIs for 2 weeks. A more recent study performed in rats demonstrated that PPIs lead to a marked exacerbation of small intestinal ulceration induced by NSAIDs, which was transferable to germ-free mice via microbiota isolated from the PPI-treated rats. This observation points to an important role for microbial alterations. When PPIs were administered alone, significant changes in intestinal microbiota were observed, with 80% reduction in the levels of the beneficial *Bifidobacteria* spp, whereas little effect was detected on the morphology of the intestinal mucosa [24].

It is generally accepted that PPI therapy can alter intestinal microbial profiles by inducing hypochlorhydria resulting in a diminished host defense against certain bacteria [25-28]. A recent study by Lombardo et al. indeed suggested that PPI therapy in humans may potentially result in small intestinal bacterial overgrowth (SIBO) [13]. It is not known whether the changes in intestinal microbiota induced by PPI therapy contribute to the development of symptoms and clinical conditions such as IBS [29]. In another recent study using duodenal aspirates, no clear association was found between SIBO with IBS or PPI use [30]. It therefore still remains unclear whether SIBO, if at all present in IBS, is a cause or merely an epiphenomenon of IBS, as microbial alterations most probably are not the sole explanation for symptom development in IBS [29]. Overall, it is tempting to assume that PPIs can potentially induce alterations in intestinal microbiota, albeit not to a clinically significant degree, which can in turn impair the capacity of the intestine to respond to noxious agents, such as NSAIDs. Such ‘two-hit’ theory could provide an explanation for the relevance of co-exposure to PPIs and NSAIDs in the onset of IBS symptoms [31].

Besides PPIs and NSAIDs, the use of SSRIs was also associated with IBS in our study, but only when patients with psychiatric comorbidity were included in the analyses. Excluding IBS patients with psychiatric comorbidities from the analyses, accounting for 38% of our study population, resulted in a loss of positive association with exposure to SSRIs. This may be due to the high prevalence of psychiatric comorbidities in this population for which IBS patients receive SSRI therapy. This observation therefore suggests that the association of IBS and SSRI use is probably due to the therapeutic application of SSRIs for preexistent psychiatric conditions. Although no data were available on the duration of psychiatric comorbid conditions, we postulate that these may have been present prior to the onset of the gastrointestinal symptoms and are therefore less likely to be involved in triggering IBS symptoms.

Although we were able to demonstrate a positive association with IBS and the use of drugs known to alter intestinal physiology, our study is hypothesis generating rather than proving an etiological relationship due to a number of limitations. While it is apparent that IBS patients utilize medication more often than controls, case–control studies generally do not allow interpretation with respect to a causal relation. In an attempt to establish a temporal relationship, we aimed to assess exposure to medication *prior* to onset of symptoms, implicating that this exposure can potentially trigger symptoms characteristic for IBS. However, we were not able to report on exact symptom history in the 180-day period investigated for drug exposure in relation to drug intake, which hinders the establishment of a true cause-effect relationship. We were also unable to report on a potential relationship between drug use and the severity of IBS-related symptoms. Furthermore, we could not take into account the use of over-the-counter medication. Also, our patient population is possibly a selected population consisting of patient presenting to our secondary/tertiary referral, which may not represent the IBS population as a whole.

## Conclusion

Our case–control study points to an increased use of certain drugs prior to consultation for symptoms in IBS patients, in particular PPIs and NSAIDs. Both PPIs and NSAIDs are frequently prescribed drugs and IBS patients are prone to over-utilize drugs. Therefore, one should be aware that prescribing PPIs for upper GI complaints or NSAIDs for pain relief may potentially trigger mechanisms resulting in symptom generation representative for IBS. Animal data suggest that the combination of PPIs and NSAIDs, in particular, is able to induce profound alterations in intestinal homeostasis. As case–control studies generally do not allow interpretation of a cause-effect relationship, further research should include prospective evaluation of PPI users and NSAIDs users monitoring the development of IBS-symptoms in relation to drug exposure to ascertain whether this increased exposure to PPIs and NSAIDs should be considered as legitimate etiological factors in IBS.

## Abbreviations

IBS: Irritable bowel syndrome; NSAID: Non-steroidal anti-inflammatory drug; PPI: Proton pump inhibitor; SSRI: Selective serotonin reuptake inhibitor; ACE: Angiotensin converting enzyme; SIBO: Small intestinal bacterial overgrowth.

## Competing interests

The authors declare that they have no competing interests.

## Authors’ contribution

DK analyzed data and wrote manuscript, GD, GM and JWK collected data, AAM supervised manuscript preparation. All authors read and approved the final manuscript.

## Pre-publication history

The pre-publication history for this paper can be accessed here:

http://www.biomedcentral.com/1471-230X/12/121/prepub
